# Notes on Feigned Insanity

**Published:** 1848-04-01

**Authors:** C. Lockhart Robertson


### NOTES ON FEIGNED INSANITY.

BY C. LOCKHART ROBERTSON, M.D.

MEDICAL STAFF, ATTACHED TO THE MILITARY LUNATIC ASYLUM AT YARMOUTH, ETC.

" King. What he spake, though it lack'd form a little,
Was not like madness." *Hamlet.*

" Edgar. While I may scape,
I will preserve myself: and am bethought
To take the basest and most poorest shape,
That ever penury, in contempt of man,
Brought near to beast: my face I'll grime with filth;
Blanket my loins ; elf all my hair in knots ;
And with presented nakedness out-face
The winds and persecutions of the sky.
The country gives me proof and precedent
Of Bedlam beggars, who, with roaring voices,
Strike in their numbed and mortified bare arms
Pins, wooden pricks, nails, sprigs of rosemary ;
And with this horrible object, from low farms,
Poor pelting villages, sheep-cotes, and mills,
Sometimes with lunatick bans, sometimes with prayers,
Enforce their charity." *King Lear.*

Section 1. History.—The earliest record we possess of mental disease being feigned, is in the fabled story of Ulysses, who is supposed to have lived 1190 years before Christ. Ulysses, on being summoned to join the Greek army in the expedition against Troy, pretended to be insane, in order not to leave his beloved Penelope. He yoked a horse and a bull together, and ploughed the sea shore, and sowed salt in the furrows instead of corn. Palamedes perceived this deceit, and exposed it by placing the infant child of Ulysses, Telemachus, before the plough of his father, when the latter turned the plough a different way, to avoid injuring the child, which appears to have satisfied the minds of the Greek princes as to his sanity.*

The next instance of feigned insanity is that related by the sacred historian† of the royal psalmist, in the following words:—"And David changed his behaviour before them, and feigned himself mad in their hands, and scrabbled on the doors of the gate, and let his spittle fall down upon his beard. Then said Achish unto his servants, Lo, ye see the man is mad: wherefore then have ye brought him to me? Have I need of madmen, that ye have brought this fellow to play the madman in my presence? Shall this fellow come into my house?"

The third instance of feigned insanity recorded in history is that of L. Junius Brutus, elected consul upon the expulsion of the Tarquins from Rome, B.C. 509. His story ran as follows:—The sister of Tarquinius Superbus, seventh king of Rome, married M. Brutus, a man of great wealth, who died, leaving two sons under age. Of these the elder was killed by Tarquin, who coveted their possessions; the younger escaped his brother's fate only by feigning idiocy, whence he is supposed to have received the surname of Brutus.‡

Section 2. CAUSES.—Insanity may be feigned,—

1. To escape from an obligation: as with the soldier, to obtain his discharge from the service.

2. To escape the punishment due to crimes committed, the criminal may feign one or other of the forms of mental disease.

It will at once be apparent how important the forming of a just diagnosis is.

Section 3. GENERAL OBSERVATIONS ON THE SUBJECT.—The discrimination between real and feigned insanity must always be attended with great difficulty; indeed, it is impossible, without a practical acquaintance with the disease, and without long-continued observation of the case in

* J. Lempriere, D.D. A Classical Dictionary. Art., Palamedes—Art., Ulysses.

† 1 Samuel, chap. xxi. ver. 13—15.

‡ The tale of Brutus having feigned idiocy, though related by most of the Roman historians, is "irreconcilable with his holding the responsible office of Tribus Celerum under Tarquinius; and that he did hold this office seems to be an historical fact, (Pompon. de Orig. Turis, Dig. 1, tit. 2, s. 2, sec. 15,) and the story of his idiocy probably arose from his surname, which is generally supposed to signify 'an idiot,' (Liv. i. 56; Dionys. iv. 67, who translates it ἠλιθιος; Nonius, p. 77.) Festus, however, in a passage which is pointed out by Arnold (Rom. Hist. i. p. 104), tells us that *Brutus*, in old Latin, was synonymous with *gravis;* which, as Arnold remarks, would show a connexion with βαρυς. The word may, therefore, as a surname, have been originally much the same as Severus. This conjecture we think more probable than that of Niebuhr's, who supposes it to mean 'runaway slave,' and connects it with the Brettii, 'revolted slaves,' whence the Brutii are supposed to have derived their name, (Strab. vi. p. 225; Diod. xvi. 15; Gell. x. 3;) he further observes, that this name might easily have been applied by the Tarquins to Brutus as a term of reproach, (Rom. Hist. i. pp. 63, 98, 515.)—*William Smith, LL.D.* Dictionary of Greek and Roman Biography, &c. Vol. i., Art., Brutus. London, 1844.

question, to arrive at a satisfactory conclusion, and truly to decide whether or not the symptoms of mental disease be feigned. "Nothing," says Ray,* "requires a severer exercise of a physician's knowledge and tact, than a case of simulated insanity." "Many of the instances on record," says Dr. Conolly,† "show the great difficulty of pronouncing a decided opinion in cases in which no man would willingly run the risk of being wrong;" and again, "where lunacy is feigned, it may be impossible to determine that it is so, without watching the patient for some time, when he does not know that he is watched, and by night as well as by day."‡ "Insanity," says Mr. Marshall,§ Deputy Inspector-General of Army Hospitals, "has been frequently feigned by soldiers who wished to obtain their discharge, and no doubt some have gained their object. But it is also true, and the fact is a melancholy one, that real insanity has been mistaken for feigned, and the patients treated and punished as impostors. Facts of this kind ought to lead medical officers to study with great care the indications of insanity—a branch of information which can only be thoroughly obtained in receptacles for the insane—and whenever there is a shadow of doubt, to proceed with the utmost caution. . . . . It is not so much because fictitious madness has been treated as real, but because real madness has been treated as fictitious, that I urge the necessity of medical officers devoting some attention to the study of this class of diseases." Dr. Isfordink|| informs us, that in the Austrian army "mental disease is frequently successfully feigned."

The acknowledged difficulties in the diagnosis of mental disease ought certainly to impress those in authority with the necessity, in doubtful cases, of being guided only by the opinion of physicians who have made mental disease their study. This has been well pointed out by Ray:¶ "The effect the difficulty of detecting attempts to feign insanity should have on the mind of those who are to form their opinions by the evidence they hear, should be to impress them with a strong sense of the necessity of an intimate, practical acquaintance with insanity on the part of the medical witness, and convince them that without this qualification the testimony of the physician is but little better than that of any one else."

Section 4. A CASE ILLUSTRATING THE DIFFICULTY OF THE DIAGNOSIS.—The following case, related by Dr. Conolly,** illustrates so forcibly the difficulty of distinguishing between real and feigned insanity, that I quote his account of it in full.

"I was a few years ago," he says, "requested to see a man confined in gaol for the crime of cutting off his wife's head. This man had made no attempt to deny the deed, or to escape the consequences. For some time after he was taken to prison his conduct was quiet, and on common subjects he would talk in a common way with his fellow-prisoners.

* A Treatise on the Medical Jurisprudence of Insanity, p. 228. Edinburgh, 1839.
† An Inquiry concerning the Indications of Insanity, p. 459. London, 1830.
‡ Ibid. p. 467.
§ The Enlisting, Discharging, and Pensioning of Soldiers, p. 161. 2nd Edition. Edinburgh, 1839.
|| Militaerische Gesundheits Polizei. Erster Bandseite 51.
¶ Op. cit. p. 230. ** Op. cit. p. 455.

When he was asked about the murder, and reminded that he would certainly be hanged for it, he always said that he did not know he had done any harm. After being confined five or six weeks, he occasionally showed a disposition to be violent, and on one occasion put a handkerchief round his neck, as if he intended to hang himself. Subsequently he became taciturn, and his demeanour changed to that of an imbecile person, which it was at the time of my seeing him. He wore a woollen cap, which he had taken from one of the prisoners, and carried a piece of wood about with him, which he represented by signs to be his sword; for he would not speak nor answer any questions, only breaking silence now and then, by repeating the word 'cabbage,' without any kind of meaning. He had buttons and other common trinkets tied round his wrist, and he had made a great many attempts to walk out of the hospital of the prison, in which he was lodged. When a watch, or any shining substance was shown to him, he would assume an idiotic smile, and begin to dance.

"*Notwithstanding all these appearances, I could not help suspecting that the man was playing a part.* The nature of his crime, and his conduct after committing it, certainly went far to support the idea of his insanity, and the insanity might have been coming on some time before the murder; and although he might be cunning, he might still be insane. Yet the mixed character of his mental disorder, and the rapid supervention of idiocy on a quiet form of insanity, in a man of thirty-five, seemed to me to be unusual circumstances. There was nothing in his manner which *might* not very easily have been the effect of imitation; and although he would not answer questions, I observed that he both heard and understood them,—at least, when I asked him, a little sharply and unexpectedly, if he did not know me, he immediately looked up, which he would not do at other times, and shook his head. I saw, too, that although he never looked directly at any one, except at that particular moment, he was in reality very watchful of their movements, even when they were distant from him. Several proofs of this occurred in a short time, and he always made a sudden run towards the door, when any one opened it to go out.

"*I mention this case exactly as it was presented to me, without attempting, even now, to say whether the man was mad or not.*"

Section 5. THE DIAGNOSIS.—Seeing, then, that the diagnosis between real and feigned insanity is attended with so great difficulty, it becomes of importance to endeavour to discover rules which may guide us in the examination of any supposed case of feigned mental disease.

There is only one broad and simple rule—viz., *an intimate acquaintance with the varied phases of intellectual and moral disorder which may affect the human mind;* and in proportion to the extent of his knowledge of this subject, will be the physician's success in deciding on suspected cases.

Certain distinctive marks which are likely to exist between a case of real and one of feigned insanity, may, however, be deduced from this knowledge.

A few such diagnostics I have, in the following section, endeavoured briefly to present, under the heads of mania, dementia, (including chronic mania,) monomania, melancholia, imbecility.

*a. Mania.*—Although mania might be simulated, so as readily to impose upon those not acquainted with the symptoms of the disease, I feel satisfied that any one conversant with the treatment of insanity, would detect the impostor.

It is a physical impossibility for a person of sound mind to present the *continued* watchfulness, excitement, and resistance to the influence of medicine, which characterize this disorder.

Again, the premonitory symptoms, as diseased action of the moral feelings, disorder of the digestive functions, headache, sleeplessness, &c., will, in a case of feigned insanity, be absent.

A careful consideration of this point, together with the continued watching of the suspected person for a day or two, and the administration of an ordinary dose of opium, tartrate of antimony, colocynth, &c., would go far to aid in forming a correct diagnosis. Further, the insensibility to all external impressions, as hunger, thirst, &c., which pre-eminently distinguishes mania from the other varieties of mental disease, as also the total absence of all sense of decency and care for cleanliness, will not readily be for any period simulated.

Violence and incoherence of thought are the only indications associated in the public mind with mania, which being present while the above-noticed premonitory and accompanying symptoms are absent, would readily enable us to detect the impostor.

The frequency of the pulse has been much insisted on as a diagnostic of mania, particularly by Drs. Rush and Foville, and the late Sir H. Halford:

> "My pulse as yours, doth temperately keep time,
> And makes as healthful music: it is not madness."—*Hamlet.*

The following table would, however, lead to the conclusion that frequency of the pulse cannot be considered as diagnostic of mania. I extract it from Professor Guy's "Principles of Forensic Medicine." The observations were made on eighty-nine insane females by Leuret and Mitivié, and on fifty healthy persons of the same sex by Dr. Guy. The results are expressed in per centage proportions of the whole number of observations, and show that *in forty-two per cent. in healthy females the pulse was above ninety, while in insane females, in only nineteen per cent. did it exceed ninety.*

| State of Pulse. | Leuret and Mitivié. | Professor Guy. | |
|---|---|---|---|
| | | Standing. | Sitting.* |
| Above 100 | 8 per cent. | 30 per cent. | 12 per cent. |
| 80 to 99 | 11 ,, | 12 ,, | 18 ,, |
| 80 to 89 | 43 ,, | 24 ,, | 20 ,, |
| 70 to 79 | 33 ,, | 22 ,, | 32 ,, |
| 60 to 69 | 4 ,, | 12 ,, | 14 ,, |
| Under 60 | 1 ,, | 0 ,, | 4 ,, |

* It being just possible that Leuret's observations were made in the sitting posture, Dr. Guy has given a column to that position also, which latter observations render the relative proportions above 90, in healthy females 30 per cent., in insane females 19 per cent.

*b. Dementia,* (*including chronic mania.*)—This disorder would be more readily feigned than mania.

Although here there is present partial incoherence of thought, the patient going off at a tangent from the subject of conversation, he generally, when questioned, is enabled to fix his ideas, and give a pertinent answer to a question put to him. Again, the perfect state of the memory of long past events, as compared with that of recent, is a striking feature in the real disease, not likely to be simulated. The impostor, in his anxiety to impress his hearers with the perfect disorder of his intellect, would, in all probability, overact his part, and give to every question an absurdly false answer.

Still, in the more aggravated forms of this disorder, the power, even for an instant, of fixing the ideas, and the memory of even past events are so entirely lost, that these points would not fail in establishing the diagnosis.

In such instances, the previous history of the case would aid much in deciding as to the reality or simulation of the disease, the symptoms of confirmed dementia not generally presenting themselves but as a sequel to mania, monomania, or some other form of mental disease. Again, such persons are insensible to the operation of the passions of hope, fear, anger, &c., the emotions of which may, in those feigning dementia, perhaps be produced. Shakspeare, who evidently must have studied insanity from nature, notices this in that beautiful delineation of feigned dementia or chronic mania in the character of Edgar,—

> " My tears begin to take his part too much,
> They'll mar my counterfeiting."—*King Lear.*

Foderé, in his " Traité de Médecine Légale," mentions having thus detected an impostor, simulating this variety of insanity—viz., by ordering the application of the actual cautery.

An American writer* coolly informs his readers, " That in the English naval and military service, where the medical officer is often called on to deal with feigned insanity, punishment is much resorted to, *on the principle that if the affection be counterfeited, it will be more efficacious than anything else in restoring the impostor to his right mind, and if real, it will do good by acting as a powerful derivative.*" Such ignorance, requires only to be noticed, carrying, as it does, its own refutation with it.

In cases of difficulty, confinement with observation, in an asylum for the insane, is our last resource, and cannot fail in the course of time in proving successful. Since the establishment of the Military Lunatic Asylum, in 1819, (where all soldiers labouring, or supposed to labour, under continued mental disease are sent,) the attempts to feign insanity, for the purpose of obtaining a discharge from the service, now seldom occur.

I have at present a case under my observation in which I suspect this variety of mental disease has been feigned with the above object. The patient at present professes to be cured of the insanity which he stated he laboured under on admission into this asylum. At that period he pre-

* Ray, op. cit., p. 242.

sented symptoms of chronic mania, talking unconnectedly, but only when he considered himself to be observed, while he exhibited a loss of memory not reconcilable with the recent invasion of the disorder, or with its extent. The threat of coercive measures sufficed to produce, in a few days, what he terms his cure. The existence, however, of a depression in the left frontal bone—the result of a kick from a horse—together with one or two minor symptoms of mental derangement, demand a longer period of observation before any decided opinion can be expressed, and aid in the illustration of the difficulty of diagnosing feigned partial insanity.

*c. Monomania.*—The simplest form of this disease is characterized by the presence of a false idea, or hallucination, which hallucination might with considerable success be simulated.

The most marked difference between a real and feigned case of monomania is in the condition of the power of reasoning. A real monomaniac cannot be reasoned out of his false ideas; and in the maintaining of them will set all the principles of logic at a defiance which the impostor would not, from a fear of discovery, venture to do. "In real monomania the patient never troubles himself to make the subject of his delusion square with other notions with which it has more or less relation; and the spectator wonders that he can possibly help observing the inconsistency of his ideas, and that when pointed out to him, he should seem to be indifferent to, or unaware of, this fact. In the simulator, on the contrary, the experienced physician will detect an unceasing endeavour to soften down the palpable absurdity of his delusions, or reconcile them with correct and rational notions."—Ray, op. cit.

Again, the impostor will endeavour to force his delusion on the notice of observers, while the real monomaniac rarely recurs to his false ideas, unless when questioned, or when the conversation bears upon the subject.

These two points appear to me to be the safest grounds on which to endeavour to form a correct diagnosis between real and feigned monomania.

The more complicated form of monomania—viz., that preceded and accompanied by perverted action of the moral powers, and in which the delusion is but a symptom of the existing moral disorder, is not likely to be feigned—still less likely to be successfully so.

*d. Melancholia.*—The simplest form of melancholia—viz., that unattended by bodily disease, and exhibited chiefly in an obstinate refusal to answer questions, and in a total disregard of all that is passing on around, might be successfully simulated. A case of this nature occurred to me, which I had under my observation for several months, and where I did not even suspect that the disorder was feigned.

In suspected cases, the endeavouring, as is recommended at page 282, to excite one or other of the mental emotions, and careful observation, are the only diagnostic marks that occur to me.

It is a disorder with which the public are not so well acquainted as with general or partial mania, and which is not, therefore, so likely to be feigned.

*e. Imbecility.*—Weakness of intellect is sometimes feigned by soldiers, and, as Mr. Marshall informs us, with success.

"Mental incapacity," he states, "or inaptitude for acquiring the manual and platoon exercise, is easily feigned, and very difficult of detection. It is very natural that regimental officers should wish to get inefficient soldiers discharged, whether the unfitness arises from physical, moral, or intellectual causes; but the general interests of the service require, that no doubtful case of disability should be recommended to be discharged, more especially on account of alleged weakness of intellect."

Mr. Marshall relates two cases in which inability to learn the drill exercises was successfully feigned; and then concludes with a statement, in the which I entirely concur, "that unless in well marked cases, where the mind is weak on all subjects, and where that weakness is expressed in the countenance, or readily discoverable during conversation, no man ought to be recommended for discharge on account of mental defects; because, if the disability is not obvious, he may re-enlist and be approved for the service."

*Royal Barracks, Yarmouth,*
*February*, 1848.

